# P-1791. Low Utilization of Methicillin-Resistant *Staphylococcus aureus* Screening in Orthopedic Surgeries

**DOI:** 10.1093/ofid/ofae631.1954

**Published:** 2025-01-29

**Authors:** Genesis S Huerta-Vera, Lauren Green, Kavya Parimi, Nabihah A Chaudhary, Megan Wein, Pamela J McCormick, Marci L Pursglove, Ricardo Arbulu

**Affiliations:** UPMC, Pittsburgh, Pennsylvania; UPMC Mercy, Pittsburgh, Pennsylvania; UPMC Mercy, Pittsburgh, PA, Pittsburgh, Pennsylvania; UPMC Mercy, Pittsburgh, Pennsylvania; UPMC Mercy, Pittsburgh, Pennsylvania; UPMC Mercy, Pittsburgh, Pennsylvania; UPMC Mercy, Pittsburgh, Pennsylvania; University of Pittsburgh Medical Center, PITTSBURGH, PA, Pennsylvania

## Abstract

**Background:**

Guidelines recommend screening for methicillin-resistant Staphylococcus aureus (MRSA) nasal colonization to identify patients undergoing orthopedic implants who may benefit from perioperative vancomycin. Culture-based MRSA screening may take days to result, yet it is the only technique available in many centers, including ours. Therefore, we investigated the proportion of cases in which MRSA screening results are available at time of orthopedic implants at our center, and explored the rationale for perioperative choice when this information is absent.

**Figure:**
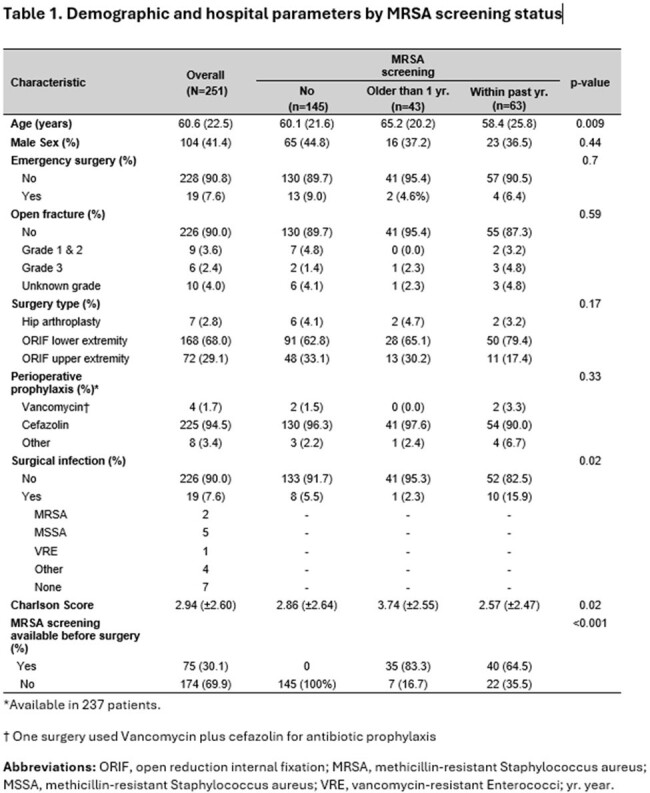

**Methods:**

We conducted a retrospective chart review of all consecutive patients 18 years or older who underwent orthopedic implant procedures for the calendar year 2023 at UPMC Mercy, Pittsburgh, PA. Patients undergoing multiple procedures within 24 hours were counted once. We report a preliminary analysis for 44% of the total population. Data was analyzed by MRSA screening status.

**Figure:**
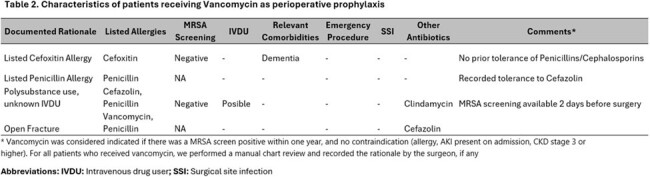

**Results:**

In 251 surgeries performed in 247 patients, MRSA screening within one year prior to surgery was available only in 63 (25.1%). An additional 43 (17.1%) had results older than one year, while 145 (57.8%) had no MRSA screening. Patients that had MRSA available within a year did not defer from those with older or absent results in the proportion of emergent surgeries, types of surgeries, or perioperative prophylactic antibiotics received (Table 1). Only 43 (17.3%) patients had MRSA screen collected during the admission.

Only four patients received perioperative vancomycin, only one had a clear indication for this choice (Table 2). Of six patients with an indication for perioperative vancomycin, none received this antibiotic.

**Conclusion:**

In patients undergoing orthopedic implants, MRSA screening results were available at time of surgery in less than half of cases, screening was infrequently ordered during the admission for surgery, and the results did not seem to affect the antibiotic choice. Non-systematic input from the three orthopedic surgeons with the highest volume at our center suggests this low utilization is due to: a. questioning the value of the screening and vancomycin prophylaxis, b. lack of systematization (of ordering, reviewing), and c. the perception that the results are unlikely to be available at the time of surgery.

**Disclosures:**

**All Authors**: No reported disclosures

